# Public Health and Preventive Medicine

**Published:** 1924-03

**Authors:** 


### PUBLIC HEALTH AND PREVENTIVE MEDICINE.

### ROCKEFELLER FOUNDATION REPORT.

THE report of the Rockefeller Foundation for the year 1922 has just been issued from 61, Broadway, New York. In addition to much other information it embodies the report of the International Health Board, from which are printed extracts in our issue of September last. The report of the President (Mr. George E. Vincent) contains some interesting observations upon medical education and the progress of preventive medicine.

### Graduate Specialists.

The idea of teaching the medical student all that is known about health and disease is on the face of it absurd (writes the President). There is complaint already that too much is being forced upon him, and that he has no time to think for himself. It is agreed that the undergraduate medical course should not seek to give a complete education but to ground the student in the fundamentals of knowledge and technique and inspire in him the scientific spirit and a sense of social obligation. These necessary limitations are resulting in the development of graduate teaching. The time seems to be coming when all the surgical and other specialities and advanced laboratory work will be taught as graduate subjects.

### Upward Tendency of Medical Education.

The raising of standards, with consequent lengthening of the medical course and the increase of its cost to both individual students and to society, gives rise to a number of serious questions. The shortening by two years of the combined elementary and secondary school period is advocated by some in order to reduce the age at which a doctor may begin his career. The granting of more scholarships to promising students is urged. There is agitation for a shorter, less expensive type of training to maintain the supply of general practitioners, on the theory that many superficially trained doctors will settle in rural districts which now lack resident physicians. Other people foresee a system of local hospitals serving surrounding areas by outpost dispensaries and visiting nurses. While some differentiation may be expected between doctors who go into general practice immediately and those who pursue graduate studies in the specialties, there is no reason to suppose that in advanced countries the standards of medical education will be lowered. The tendency at present is in quite the opposite direction.

### Importance of Laboratory Tests.

The strengthening of medical schools and emergency aid for medical scientists have a direct bearing upon the essential task of preventing disease, which is one of the leading ideals of scientific medicine. The dependence of this movement upon the knowledge, skill, and social spirit of the medical profession is too generally overlooked. Statistics of births, deaths, and sickness furnish the facts by which public health policies and procedures are guided. The data are supplied almost exclusively by practising physicians. If they are competent diagnosticians and conscientious in making reports, the resulting statistics are trustworthy; otherwise they are incomplete and misleading. If doctors are familiar with modern laboratory tests, they may not only safeguard their individual patients, but by prompt notification of contagious diseases protect the community. The extent to which a public diagnostic laboratory is utilised is one index of the intelligence, alertness, and social-mindedness of the profession in the area which the laboratory serves.

### Pioneers of Preventive Medicine.

It is immensely to the credit of the profession that doctors have been among the pioneers and leaders in the development of preventive medicine. They had the imagination and faith to realise that the chief purpose of medicine must be to keep people well, rather than to rest content with alleviating or curing diseases which might have been avoided. During the last fifty years scientific medicine has discovered the causes of many maladies and has learned how to protect individuals and communities against them. Many diseases formerly dangerous may now be discovered in their earliest stages and effectively controlled. Then, too, knowledge about the normal conditions of healthy living has so increased that people may be measurably helped to maintain sound minds in sound bodies. Furthermore, practical methods of applying science to disease-prevention have been elaborated, so that not only striking reductions in death-rates but other evidences of positive well-being have been manifested.

### The Training of Health Staffs.

Emphasis upon the preventive side of medicine in medical schools is gradually changing the attitude of the medical profession as a whole, but it cannot turn out public health administrators ready to head city and State departments of health. The too prevalent idea that any practising physician is capable of discharging the duties of a health officer needs to be vigorously combated. Such a post ought to be filled by a person who, in addition to a basic medical preparation, has had specialised training for what has become a distinct profession. This training includes lecture courses and laboratory and field work in the causes of contagious diseases and methods of controlling them, in sanitary engineering, vital statistics, administration, and other subjects. Only recently have special schools been organised to give training of this kind. The leaders in the public health movement have been doctors with sufficient imagination, character, and devotion to train themselves by the often wasteful method of trial and error. So little satisfied are they with the school of experience that they welcome heartily the new institutions.

